# Supplementary material for: Multifunctional resonant wavefront-shaping meta-optics based on multilayer and multi-perturbation nonlocal metasurfaces
Source: Light Sci Appl. 2022 Aug 3;11:246. doi: 10.1038/s41377-022-00905-6 (PMC9349264; doi:10.1038/s41377-022-00905-6)
Supplement: Supplementary file 1 — Supporting Information [file 41377_2022_905_MOESM1_ESM.docx]

Supplementary Information for

Multifunctional Resonant Wavefront-Shaping Meta-Optics Based on Multilayer and Multi-Perturbation Nonlocal Metasurfaces

**Authors:** Stephanie C. Malek^1^, Adam C. Overvig^1,2^, Andrea Alù^2,3^, and Nanfang Yu^1,*^

**Affiliations:**

^1^Department of Applied Physics and Applied Mathematics, Columbia University, New York, NY 10027, USA.

^2^Photonics Initiative, Advanced Science Research Center, City University of New York, New York, NY 10031

^3^Physics Program, Graduate Center, City University of New York, New York, NY 10016

**This PDF file includes:**

Supplementary Sections 1-12

Supplementary Figs. S1 to S19

Supplementary Tables S1 to S3

**Section 1: Review of Theory for Symmetry-Protected q-BICs**

In this section, we highlight and review a few relevant key points from our previous theoretical works on symmetry-protected q-BICs^1,2^. Our metasurfaces are based on meta-units with a dimerizing perturbation that doubles the period in real space in one spatial direction. **Figure S1b** show a perturbed meta-unit (used for the devices in **Fig. 2** in the main text) with a corresponding unperturbed meta-unit shown in **Fig. S1a**. Doubling the period in real space halves the period in k-space, which effectively folds the bands. The calculated bandstructures for the unperturbed and perturbed structures are shown in **Figs. S1c** and **S1d** respectively. In the unperturbed structure, this mode is under the light line and therefore a guided mode that does not radiate to free space. In the perturbed structure, when the bandstructure is folded, the mode is no longer a bound mode and can be excited by free space light—including by normal-incident light at the Γ point.

**
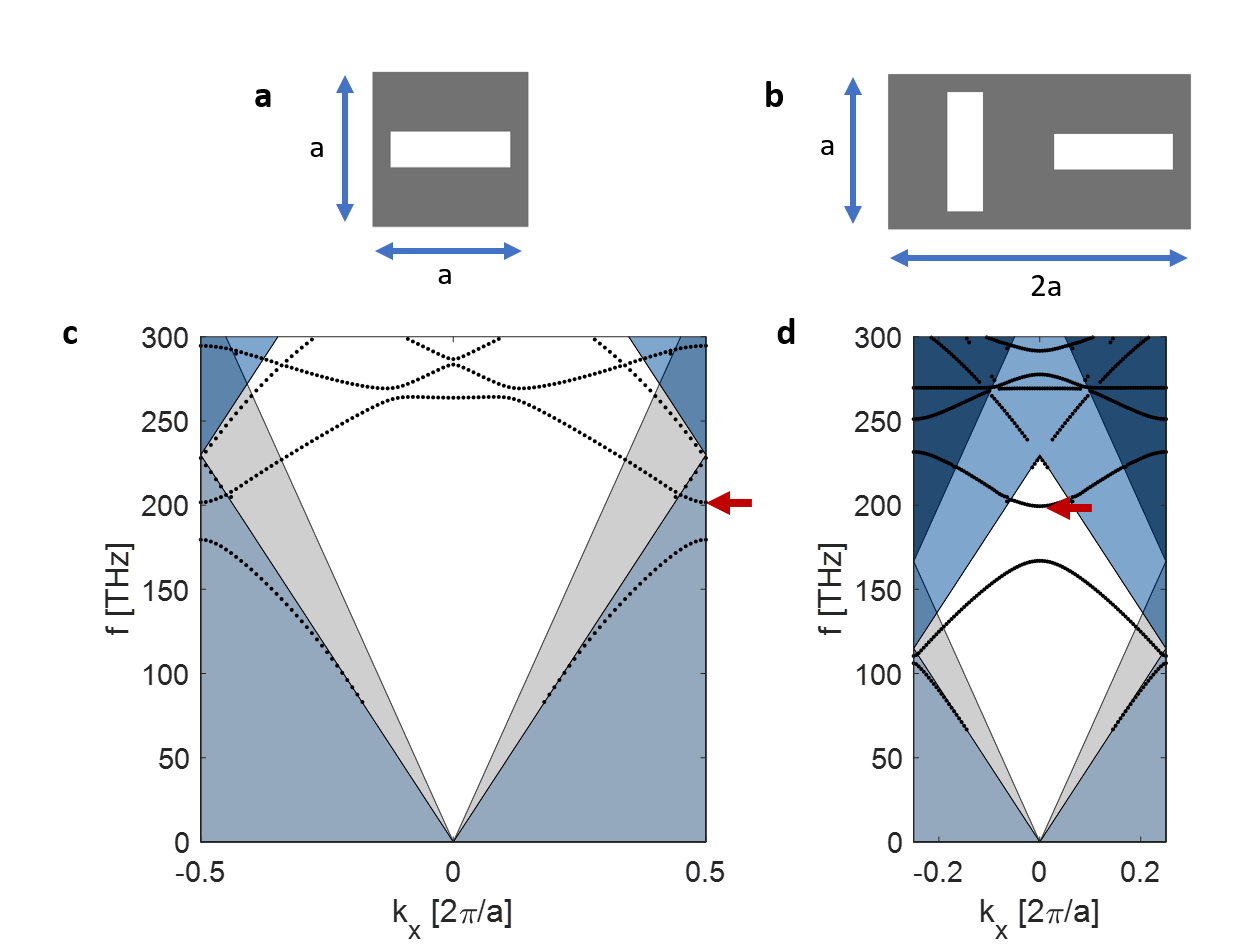
**

**Fig. S1** Illustration of dimerizing perturbations and band folding. Schematic of unperturbed (a) and perturbed (b) meta-units. The meta-unit has a dimension of a= 450 nm; the rectangular apertures have a dimension of (L-$\delta$) × (L+$\delta$) = 125 nm × 375 nm and are etched in a 125-nm silicon thin film on a glass substrate. Calculated band structures for unperturbed (c) and perturbed (d) meta-units. Red arrow indicates the mode of interest for the metasurface in **Fig. 2** of the main text.

The symmetry of the perturbed meta-unit dictates what polarization is able to excite each of these modes. Briefly, the symmetry of the perturbed portion of the mode must be the same as the symmetry of incident polarization in order to be excited by a given polarization state. We have previously developed an exhaustive catalog of selection rules with group theory^2^, and will review in the following passages the portion of selection rules most relevant to enabling geometric phase in nonlocal metasurfaces based on square lattices. Here, we consider modes with the irreducible representation A_1_ shown in **Fig. S2a**, but note there is a separate and comparable set of selection rules for modes with the irreducible representation B_1_. We consider 3 plane groups: *pmm*, *pmg*, and *p2*. The *p2* plane group can be considered a ‘child’ group of ‘parent’ groups *pmm* and *pmg* because the *p2* group has the shared symmetry operations of its parent groups, resulting in correlated selection rules. The structures from the *pmm* and *pmg* plane groups can support A_1_ modes for x- and y- polarized incident light, respectively (**Fig. S2c**). Symmetry dictates that the in-plane rotation angle α of the structures (**Fig. S2b**) is 0° for the *pmm* structure and 45° for the *pmg* structure. The *p2* plane group can also support A_1_ modes for some angle ϕ and can support any value of in-plane rotation angle α. From the *pmm* and *pmg* structures it is clear that ϕ is rotated by 90° (from x-polarized to y-polarized) when α is rotated by 45°. For this reason, we deduce that ϕ follows α as $\phi\sim2\alpha$. This means that the in-plane orientation angle α controls what polarization of light can excite q-BICs with A_1_ mode symmetry.

This relationship between orientation angle and polarization angle enables the device to support geometric phase for circularly polarized light. There are two instances of polarization conversion: conversion of free-space light into the linearly polarized mode with polarization angle $\phi\sim2\alpha$ and conversion of the linearly polarized mode back into circularly polarized free-space light. Each instance of polarization conversion imparts a phase of 2α. Approximately half of the incident power is reflected and half is transmitted because only half the light, the light polarized along 2α, is coupled to the q-BIC. The geometric phase for transmitted and reflected light is as follows:

$\Phi_{p}^{t}=\Phi_{c}^{r}=0$ (S1a)

$\Phi_{c}^{t}=\Phi_{p}^{r} =4\alpha$ (S1b)

where *t* and *r* denote transmitted and reflected light respectively, and *c* and *p* denote light of converted and preserved handedness of circular polarization^2^. So, transmitted light of converted handedness and reflected light of preserved handedness experience a geometric phase of 4α but only for the spectrally narrowband q-BIC modes. This geometric phase is double the typical geometric phase in local metasurfaces only because the correspondence between α and ϕ is doubled compared to that of a local birefringent meta-unit.

**
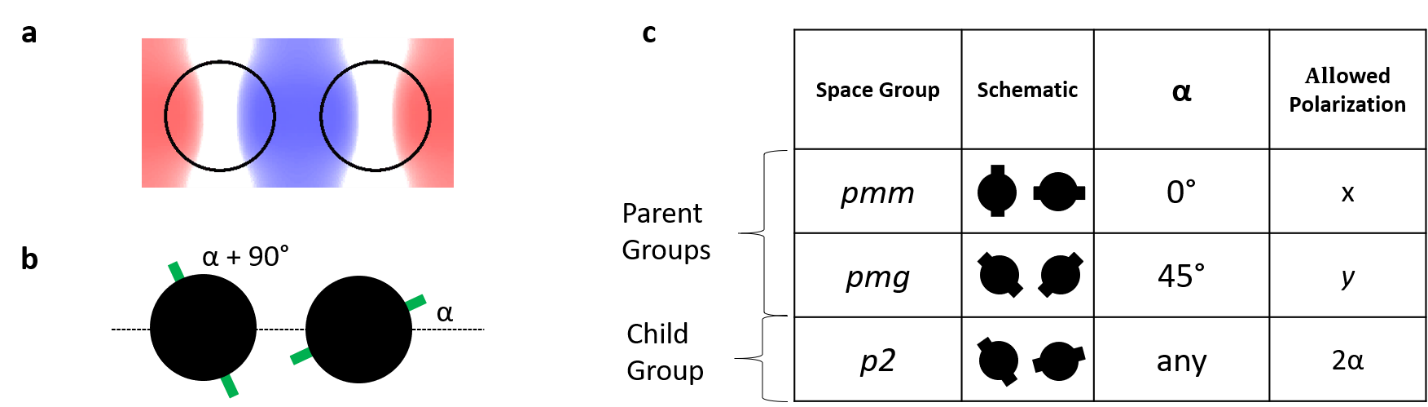
**

**Fig. S2** Illustration of the q-BIC selection rules that enable geometric phase. (a) Schematic of a q-BIC mode with an A_1_ irreducible representation. (b) Schematic relating *pmm*, *pmg*, and *p2* space groups. The green area is the perturbation and can be rotated. (c) Abbreviated table of selection rules for *pmm*, *pmg*, and *p2* space groups.

**Section 2: Meta-Unit Library Design**

In this work, we constrain ourselves to a platform of amorphous silicon (a-Si) on glass, near-infrared wavelengths, and q-BIC metasurfaces with *p2* plane groups perturbed from a square lattice. The meta-unit library used in the metalens in **Fig. 2** of the main text is shown in **Fig. S3**. **Figure S3a** shows that the meta-unit of a *p2* plane group consists of two rectangular holes with in-plane rotation angles $\alpha$ and $\alpha+90^{\circ}$, defined in the a-Si thin film. The library consists of meta-units with different $\alpha$ but otherwise identical geometrical parameters (i.e., periods A and 2A along x and y directions, respectively, and fixed dimensions for the rectangular apertures). Our FDTD simulations confirm that the phase shift of transmitted light with converted handedness of circular polarization on resonance varies approximately linearly as a function of the in-plane orientation angle $\alpha$, following $\sim4\alpha$ (**Fig. S3b**). A phase-gradient metasurface can be constructed by progressively changing $\alpha$ in a 2D array of the meta-units (**Fig. S3c**). The simulated transmission spectra of the phase-gradient metasurface shows a resonant peak for light of converted handedness and a corresponding dip for light of unconverted handedness (**Fig. S3d**). Simulated optical wavefronts of the device show that only on resonance and only for light of converted handedness the outgoing wavefront is tilted by the phase gradient (**Fig. S3e**). Notably, off resonance there is minimal transmission of converted light and high transmission of unconverted light.


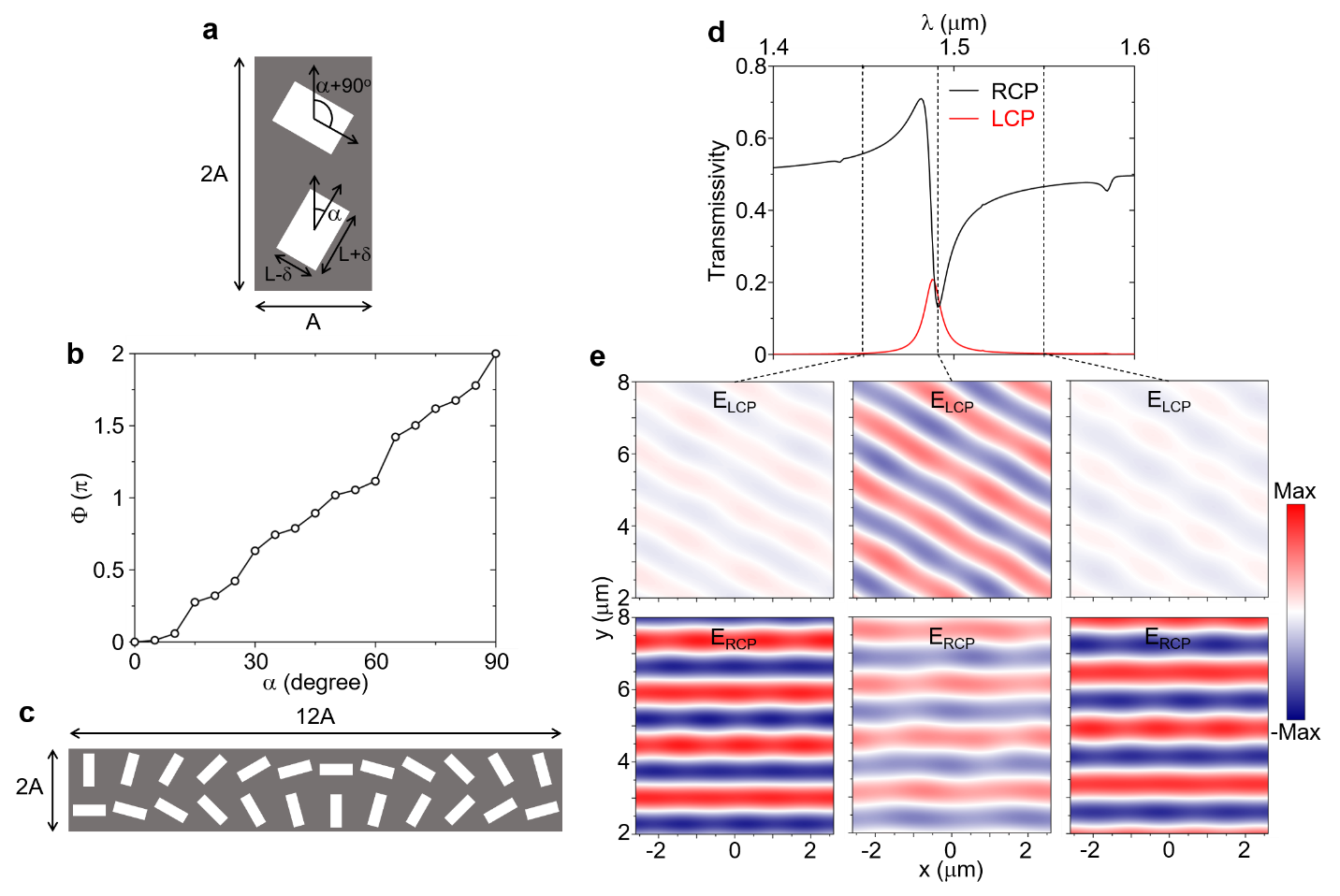


**Fig. S3** Design of a meta-unit library for nonlocal, wavefront-shaping metasurfaces and a resonant phase-gradient metasurface. (a) Schematic of a meta-unit of a *p2* plane group, generated by applying a dimerization perturbation of magnitude $\delta$ to a square lattice of square apertures. (b) Simulations showing that the geometric phase Φ of light of converted handedness of circular polarization is approximately four times of the orientation angle $\alpha$ of the dimerizing perturbation. In this specific example, the meta-unit has a dimension of A × 2A = 450 nm × 900 nm; the rectangular apertures have a dimension of (L-$\delta$) × (L+$\delta$) = 125 nm × 375 nm and are etched in a 125-nm silicon thin film on a glass substrate. (c) Schematic of a super-period of the resonant phase-gradient metasurface, consisting of 12 meta-units with spatially varying $\alpha$. (d) Simulated transmission spectra of the phase-gradient metasurface for light of the converted (red) and unconverted (black) handedness of circular polarization. The converted LCP light has a resonant peak with a Q-factor of ~130. (e) Simulated far-field electric-field profiles of the resonant phase-gradient metasurface, showing that beam steering (to a ~33.7^o^ angle) only occurs on resonance for light of converted handedness, and that the device remains largely transparent for non-resonant light.

We have three primary design goals for our resonant geometric-phase meta-unit library: (1) large spectral separation between resonant modes, (2) intermediate Q-factors (i.e., 100-500), and (3) minimal resonant wavelength dispersion. The large spectral separation between modes allows for a clear experimental demonstration of the contrast between device performance on and off resonance. The intermediate Q-factors alleviate stringent requirements on small resonant wavelength dispersion and high etch fidelity during device fabrication; Q-factors of 100-500 also match with the linewidth of our supercontinuum light source filtered by a monochromator, which is dλ~5 nm. Intermediate Q-factors and small resonant wavelength dispersion (through bandstructure engineering) together can enable the demonstration of high numerical aperture (NA) nonlocal metalenses.

In an unperturbed lattice of apertures in a photonic crystal slab (PCS), the boundary conditions of electromagnetic fields dictate that large photonic bandgaps (and thus large spectral separation between low-order resonant modes) arise when the height of the slab is small with respect to the period of the lattice^3^. This is confirmed in **Fig. S4a**, which shows the transmitted light with converted circular polarization for PCSs of rectangular holes etched into a film of silicon with the same in-plane dimensions but varying thicknesses. We use a relatively thin layer of 125-nm a-Si for its large spectral separation between low-order modes. We subsequently adjust the perturbation strength (i.e., aspect ratio of the rectangular apertures) to tune the Q-factor (**Fig. 2f** of the main text). As Q-factors vary inversely with the perturbation strength squared, more strongly anisotropic apertures produce lower Q-factors.


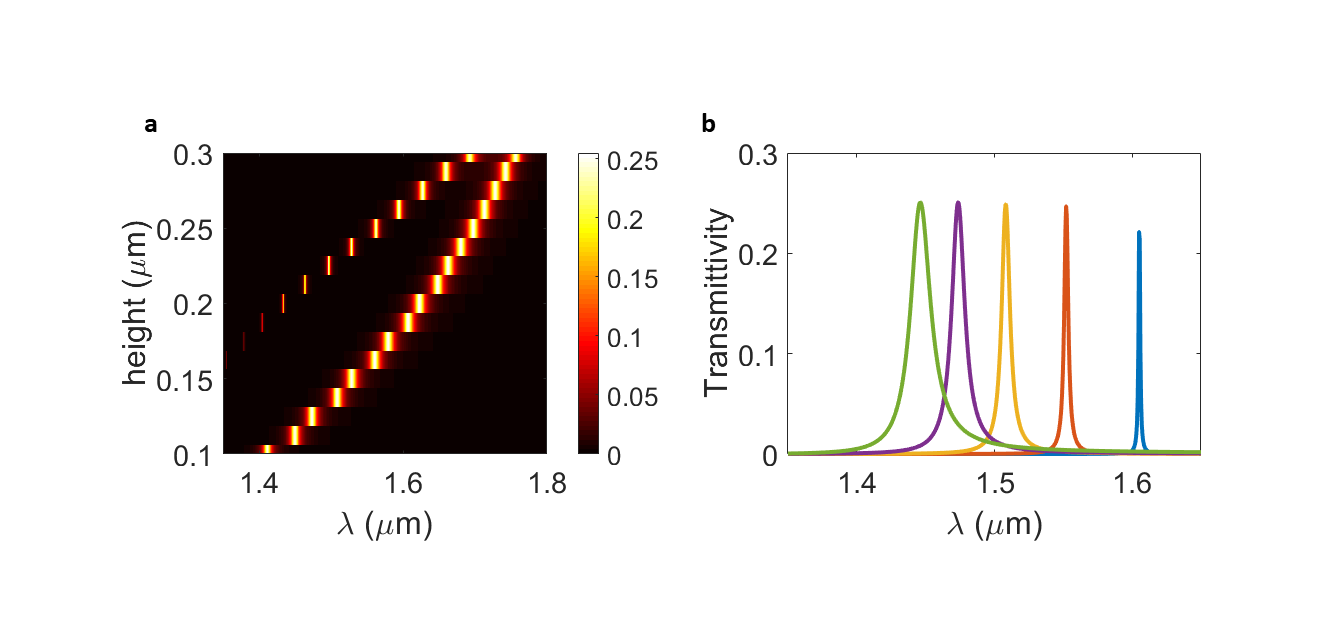


**Fig. S4** Transmitted light with converted handedness of circular polarization of a periodic array of identical meta-units composed of rectangular apertures in a-Si with dimensions of A=450 nm, L=250 nm, δ=125 nm, and varying a-Si film thickness. A schematic of the meta-unit and definitions of A, L, and δ are shown in **Fig. S2a**.

The final important goal for the meta-unit library design requires consideration of the bandstructure or resonant wavelength dispersion, i.e., the resonant wavelength as a function of the deflection angle or magnitude of the phase gradient. The meta-units themselves have a bandstructure (**Fig. S1**, **Figs. S5a** and **S5b**) such that the resonant wavelength is dispersive with incident angle. Due to the spatially varying coupling to the quasi-BIC, the resonant mode of the device in **Fig. S3c** is a supermode^2^ with a period of 12A and a Bloch wavevector of π/(6A). This additional Bloch wavevector (due to the encoded geometric phase gradient) has the important consequence of shifting the resonant wavelength according to the bandstructure of the nonlocal mode so that the band is no longer symmetric about normal incidence (**Fig. S5c**). Illuminating such a device with off-normal incident light will change the transmission angle compared to normal incident light following the generalized Snell’s law as both the incident angle and the wavelength—but not the phase gradient imparted by the device—are changed when the device is excited at an angle. The resonant wavelength is dispersive with the incident angle because of the curvature of the bandstructure of the resonant metasurface; by reciprocity, the resonant wavelength at normal incidence is also dispersive with the magnitude of the phase gradient. This represents an important design constraint for our nonlocal metasurfaces: If the dispersion of the quasi-BIC mode is too large, the device will not resonate to the optical excitation with normal incidence across its entire footprint.

**
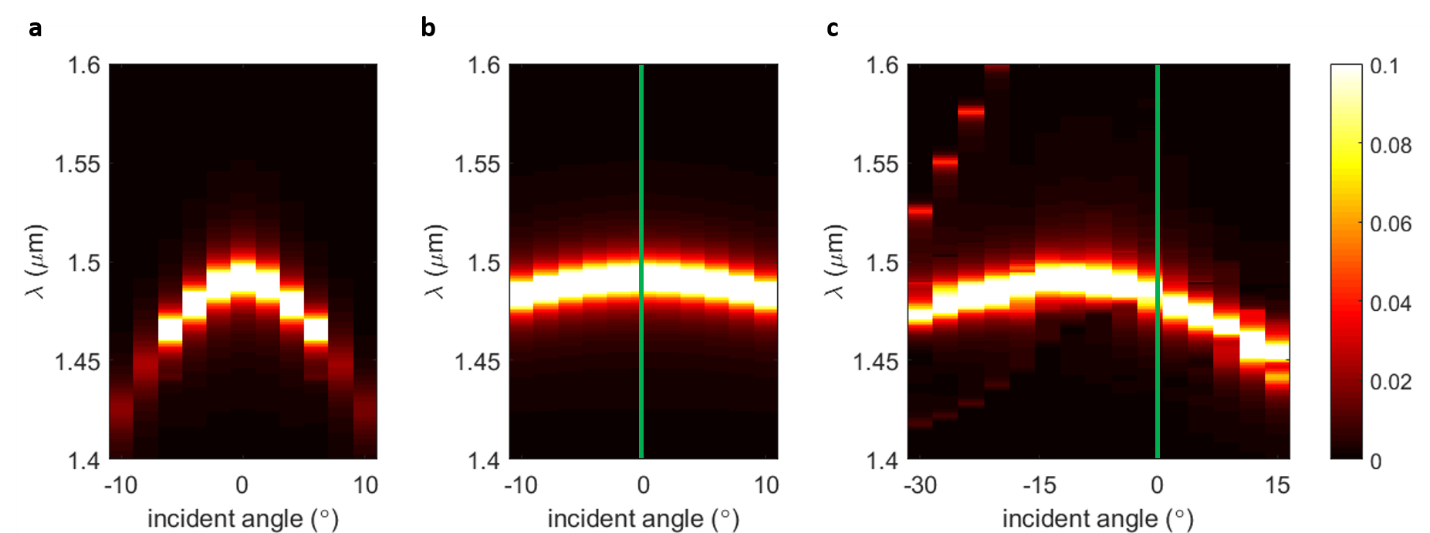
**

**Fig. S5** Dependence of resonant wavelength on incident angle for the *p2* meta-unit library in **Fig. 2** and **S3**. (a,b) Transmitted light of converted handedness as a function of incident angle for a periodic meta-unit (a) along the direction of the perturbation and (b) orthogonal to the direction of the perturbation. (c) Transmitted light of converted handedness as a function of incident angle (along the direction of the phase gradient) for a phase-gradient metasurface with a super-period in **Fig. S3c**. Green line highlights normal incident light.

Resonant wavelength dispersion can be minimized by bandstructure engineering. The achievable numerical aperture (NA) of a metalens (quantifying the range of optical deflection angles supported by the device) is limited by the resonant wavelength dispersion and the Q-factor of the resonance: The total shift in resonant wavelength from the center to the edge of the metalens should be no larger than the full width at half maximum of the resonance, requiring

$$\mathrm{NA}^{2}\leq\frac{\omega_{o}}{\left| b \right|Q{k_{o}}^{2}} (S2)$$

where *b* approximates the resonant frequency dispersion at the Γ point in k-space such that $\omega_{res}\approx\omega_{o}+bk^{2}$and $k_{o}$ is the free-space wavevector. This implies that maximizing the NA of a nonlocal metalens requires minimizing the factor $\left| b \right|Q$, achievable through flat bands and/or large perturbations.

To estimate the achievable NA of metalenses constructed from this library, we calculate the transmission spectra of several phase-gradient metasurfaces each anomalously refracting light to a different angle. We consider phase gradients arranged either parallel to or orthogonal to the dimerization direction of the dimerization perturbation (inset to main text **Fig. 2k**). For the particular meta-unit library described in **Fig. S3**, a cylindrical lens with the phase gradient orthogonal to the dimerization perturbation direction could in principle support NA larger than 0.6, whereas one with the phase gradient along the direction of the perturbation could not support NA larger than ~0.26 (main text **Fig. 2k**). The highest NA achievable for a radial lens is therefore ~0.26, limited by the most dispersive phase gradient direction. More sophisticated engineering can produce flatter bands, or smaller $\left| b \right|$, to achieve high Q-factors and high NA simultaneously. A perturbed PCS with a hexagonal lattice geometry and a *p2* plane group may also exhibit a more isotropic angular dispersion.

**Figure S6** shows the meta-unit library of the cylindrical metalens that is cascaded with the radial metalens in **Fig. 3** of the main text. The resonant wavelength of the cylindrical metalens is blue-shifted compared to that of the radial metalens so that the devices can be cascaded without their q-BIC modes overlapping spectrally. The smaller in-plane dimensions of the meta-units for the cylindrical lens blueshift the resonant wavelength.


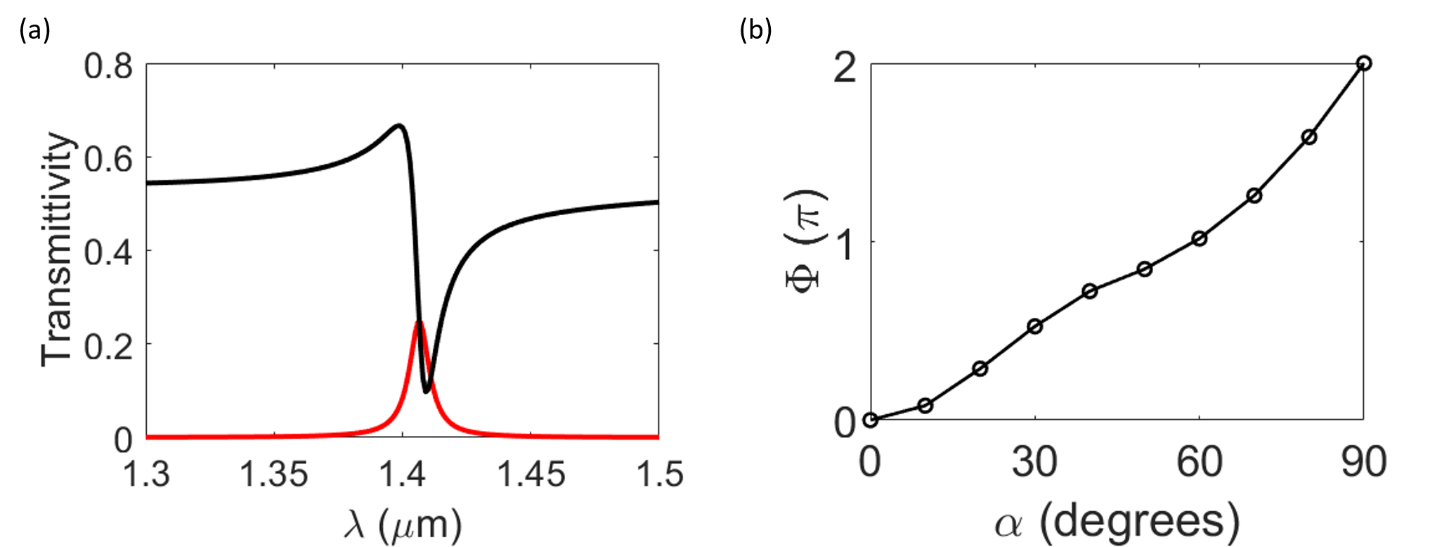


**Fig. S6** Meta-unit library for the cylindrical lens in **Fig. 3** of the main text. Each meta-unit has dimensions of A=410 nm, H=125 nm, L+δ=350 nm, and L-δ=100 nm. A schematic of the meta-unit and definitions of A, L, and δ are shown in **Fig. S3a**. (a) Transmission spectra of handedness converted (red) and unconverted (black) circularly polarized light for a periodic array of meta-units with the orientation angle of the apertures α=30°. (b) Geometric phase for converted light at λ=1.418 μm as a function of α.

**Section 3: Decay of Quasi-BIC Mode upon Excitation by Near-field Dipoles for Periodic Meta-Units and Phase-Gradient Metasurfaces**

In our nonlocal metasurfaces, q-BICs are modes accessed through free-space excitation (e.g., a plane wave). However, the physics underlying the free-space response can be broken down into two processes: (1) the coupling into the mode and then (2) the coupling out of the mode. In linear, passive, reciprocal devices (as we study here), the coupling coefficients of these two processes from each external channel (e.g., diffraction order, polarization state) must be equal^4^. Hence, in addition to studying the response to incident plane waves (as used in **Supplementary** **Section 2**), we may instead populate the q-BIC in the near field (via judiciously placed dipole sources) and observe the decay to the substrate and superstrate. This process adds insight to the underlying functionality, clarifying that the LCP and RCP components of a progressively rotating linear polarization state decay to tilted wavefronts. This tilt in the wavefront is the phase gradient upon coupling out of the q-BIC; since coupling in is reciprocal to coupling out, we readily see that external light experiences two factors of the geometric phase gradient, according to the handedness of the in-coupling and out-coupling light.

To demonstrate a mode with leakage to a controlled polarization angle, and to aid the understanding of the operation of these nonlocal metasurfaces more generally, we study the decay of the metasurfaces with dipole excitation in the near field. To this end, two magnetic dipoles per meta-unit are placed oriented vertically at the high symmetry locations of the device, with magnetic moments $m_{0}$ and ${-m}_{0}$ to match the phase profile of the mode. As shown in **Figs. S7a,b** as green markers, they are placed where the q-BIC’s modal field profile has a maximum and minimum, and due to this resonant excitation, emission upwards and downwards shows narrowband Purcell enhancement (**Fig. S7c**). The field profiles in **Figs. S7a,b** are the q-BIC mode under study, and the choice of the sign and placement of the magnetic moments evidently match the modal profile, facilitating excitation of this mode.


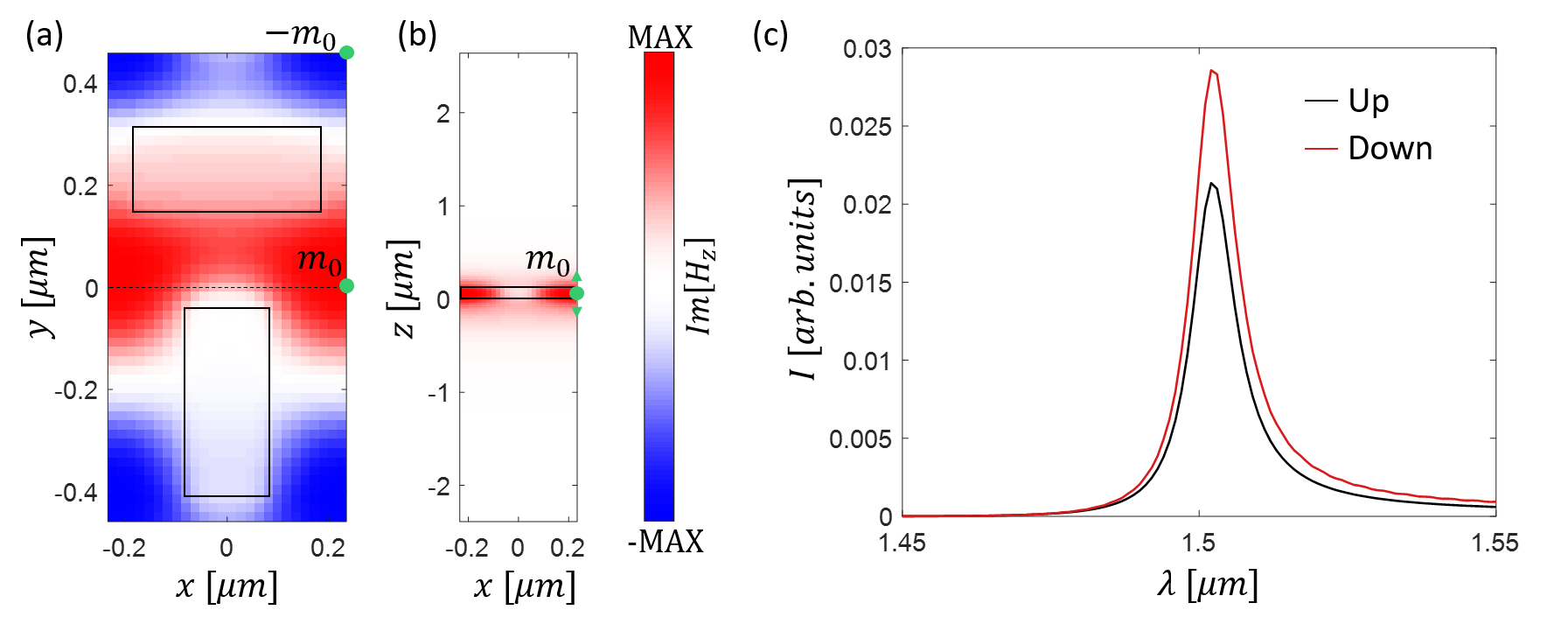


**Fig. S7** Geometry for studying the decay of a q-BIC mode upon excitation by near-field dipoles. (a,b) Resonant modal profiles overlaid with geometry (black rectangles), calculated via finite-difference time-domain (FDTD) simulations. The mode is excited by magnetic dipoles of strength $m_{0}$ (shown in green markers). The black dashed line in (a) marks the location of the xz cut in (b). (c) Intensity of light emitted upwards and downwards, showing the resonant nature of the structure near the operating wavelength.

Having demonstrated that this method excites the q-BIC mode as expected, we next study the scattered field as the angle $\alpha$ of the rectangular apertures of the meta-unit is varied. **Figure S8** visualizes the x-polarized, y-polarized, LCP, and RCP field components scattered upwards and downwards for individual meta-units used in the phase-gradient metasurface in **Fig. S3**. For instance, **Fig. S8a** shows the real part of $H_{y}$, proportional to the x-polarized electric field component, of the region external to the slab (which is located near z = 0), and the out-of-plane component of the mode near the slab, ${iH}_{z}$. The factor of $i$ is chosen so that the imaginary part of $H_{z}$ relative to the dipole excitation (which are real-valued) is chosen; that is, at this instance, the dipole sources have zero magnitude, allowing us to visualize the excited mode only. Comparing across each panel in **Figs. S8a,b**, we see that the scattered x-polarized and y-polarized components vary in both sign and magnitude. For example, when $\alpha=0^{\circ}$ (the leftmost meta-unit), the selection rules constrain the scattered light to be $H_{y}$, while when $\alpha=45^{\circ}$ (the 4^th^ meta-unit from the left), they constrain it to be $H_{x}$. Now combining **Figs. S8a,b**, we see that the polarization angle rotates as predicted across all linear polarization angles.

**
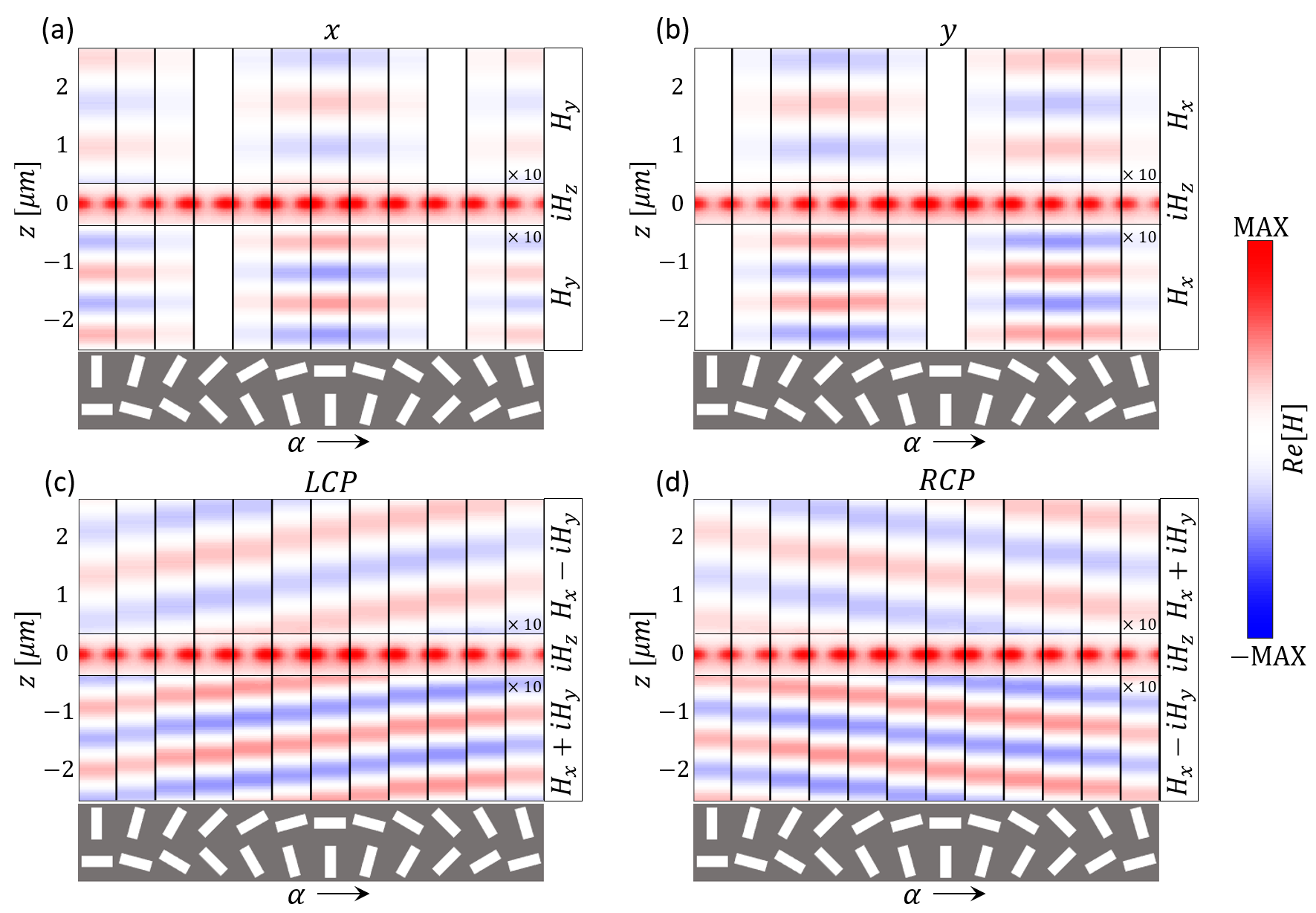
**

**Fig. S8** Decay fields of individual meta-units constituting the phase-gradient metasurface in **Fig. S3**. The in-plane components of the magnetic field are shown for x-polarized, y-polarized, LCP, and RCP light in (a), (b), (c), and (d), respectively. Each panel shows the xz cross-section of the leaky mode supported by a meta-unit, and $\alpha$ varies from 0 to 180^o^ from the leftmost to the rightmost meta-unit. In (c) and (d), the complex conjugate for the circularly-polarized field components in the downward direction accounts for the reversal of propagation direction.

Since these meta-units are designed to impart geometric phases, we next compute the LCP and RCP components of the external field, again leaving the region near the slab to display the q-BIC modal field. **Figures S8c,d** visualize the LCP and RCP scattered waves. Here, we see that each circularly-polarized component scatters with approximately constant amplitude as $\alpha$ varies, but that the phase varies across 2π. **Figure S8** therefore demonstrates the geometric phases encoded into the scattering coefficients of the q-BIC meta-units.

We may also excite a phase-gradient nonlocal metasurface and study the spatial decay of the nonlocal mode, and compare it to the behavior of its constituent meta-units. This is carried out by arraying both the meta-units in **Fig. S8** and the magnetic dipoles shown in **Fig. S7**. **Figure S9** shows the x-polarized, y-polarized, LCP, and RCP field components for the phase-gradient metasurface studied in **Fig. S3**. The x-polarized and y-polarized components vary in both sign and magnitude along the device, while in the LCP and RCP basis planewaves are observed to leak from the device with a tilted wavefront corresponding to the geometric phase gradient encoded by the device. Comparison to the results in **Fig. S8** shows excellent agreement, validating the concept of adiabatically controlling a nonlocal mode by applying the selection rules locally.


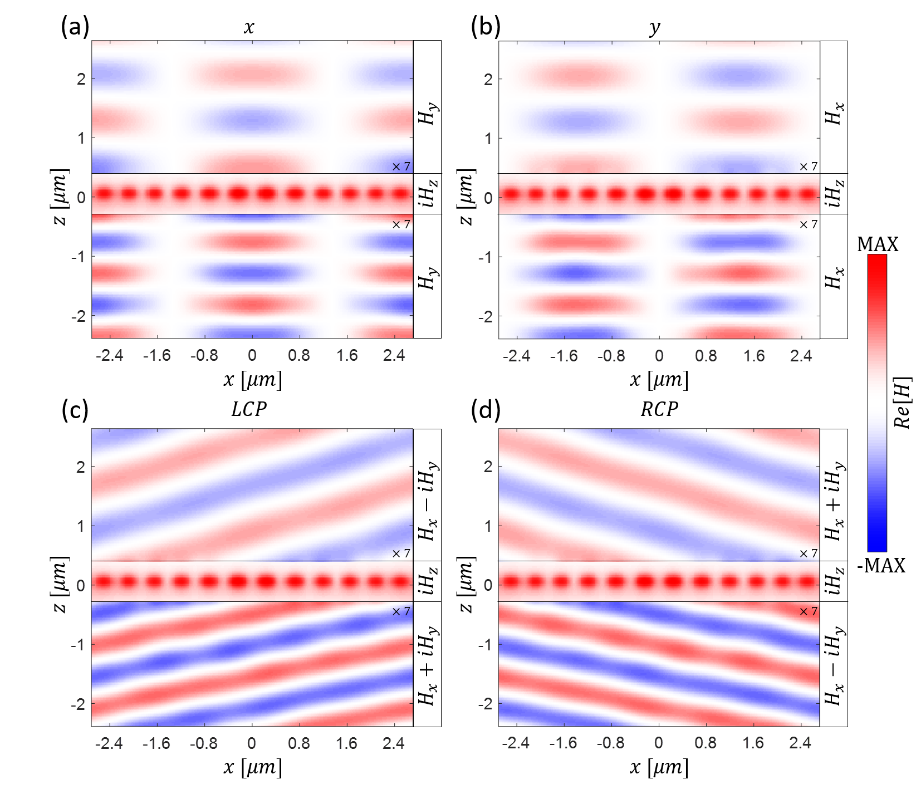


**Fig. S9** Decaying fields of the phase-gradient metasurface in **Fig. S3**. The in-plane components of the magnetic field are shown for x-polarized, y-polarized, LCP, and RCP light in (a), (b), (c), and (d), respectively. The LCP and RCP fields are, by design, planewaves tilted according to the spatial distributions of geometric phase.

**Section 4: Unpolarized Nonlocal Metalens Spectra**

**
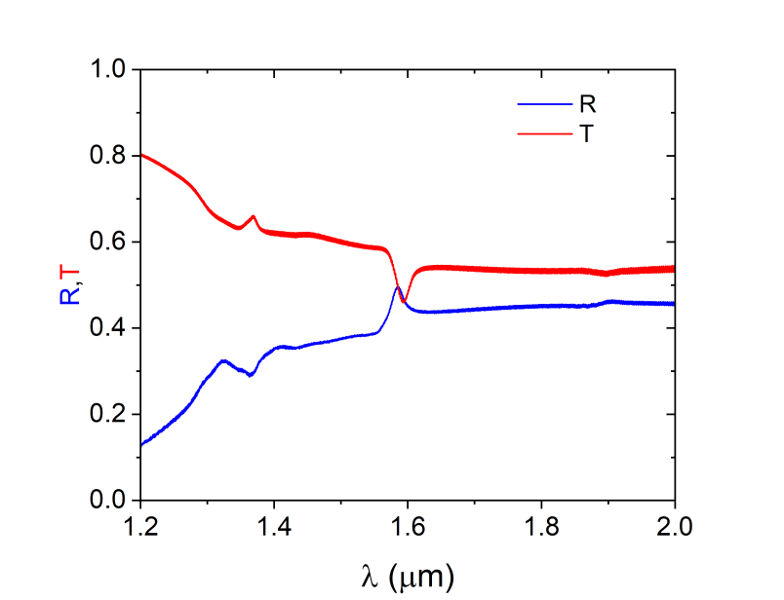
**

**Fig. S10:** Unpolarized transmission (red) and reflection (blue) spectra for the device in **Fig. 2** of the main text.

**Section 5: Quasi-Radial Lens Design**

The resonant wavelength dispersion for a phase gradient aligned along the direction of dimerization perturbation applied to a square lattice is often the primary factor limiting the achievable NA of resonant radial metalenses (**Fig. 2k**). To circumvent this limitation, we consider an alternative scheme for creating radial lenses such that the phase gradient is always orthogonal to the dimerization direction and therefore exhibits less angular dispersion of the resonant frequency. Specifically, a quasi-radial lens is created by assembling eight slices of a cylindrical lens such that each slice is the cylindrical lens rotated by an incremental angle of 45^o^ and that the phase gradient is aligned orthogonal to the perturbation direction in each slice (**Fig. S11a**). This design also considers the geometric phase accumulated by rotating the cylindrical-lens slices. Rotating a cylindrical-lens slice by an angle of $\theta$ and considering a meta-unit with in-plane rotation angle α of its rectangular apertures, the polarization angle $\phi$ of the q-BIC mode that is excitable from free space is

$$\phi\approx2\alpha+\theta. (S2)$$

The geometric phase of this meta-unit is then$2\phi$. Therefore, to form a quasi-radial lens, the in-plane rotation angle of the rectangular apertures in each section of the lens must be adjusted to account for the angular orientation of the lens slice. The adjusted in-plane rotation angle $\alpha^{'}$ can be expressed as

$$\alpha^{'}=\alpha-\frac{\theta}{2}. (S3)$$

A schematic of the resulting quasi-radial lens in **Fig. 4b** of the main text is shown in **Fig. S10b**.

**
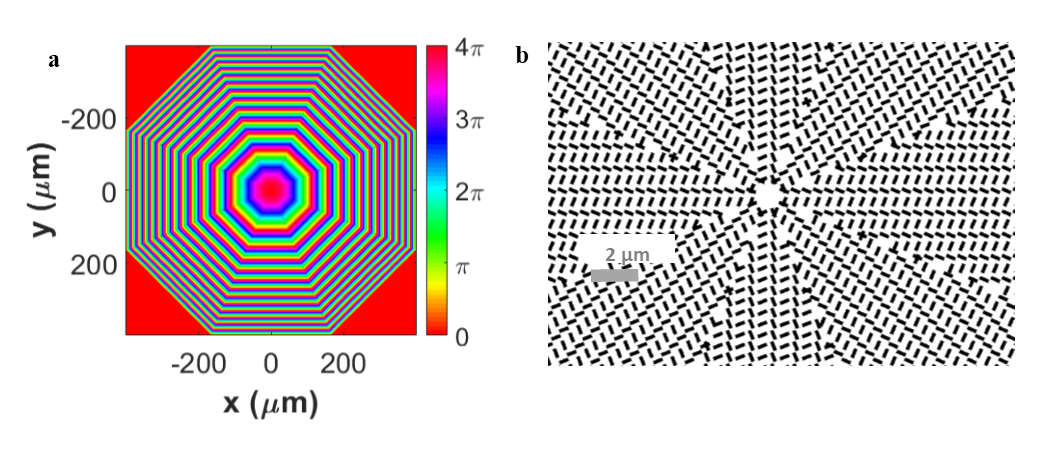
**

**Fig. S11** Design of a quasi-radial metalens. (a) Phase distribution and (b) meta-unit layout for the metalens.

At each of the eight boundaries between the cylindrical-lens slices, the device does not strongly support a q-BIC mode as a Fano resonance cannot build right at the boundaries. Here, light cannot couple into the q-BIC, so it is simply transmitted without polarization conversion or phase discontinuities. Therefore, there is no wavefront shaping from the boundaries. We note that the direction of the traveling wave within our device (the q-BIC mode has a nonzero group velocity) is parallel to the direction of phase variation and 22.5° from the orientation of the boundaries. As such, the boundaries cause less disruption to the device performance than if the traveling wave direction were perpendicular to the interface. As the interfaces comprise a small portion of the device, we expect that they do not cause substantial disruption to the wavefront shape. In principle, there is flexibility to optimize the number of sections as there are tradeoffs: Fewer sections will lead to fewer interfaces but a poorer approximation to a radial lens; more sections will lead to the traveling wave direction to be closer to parallel to the boundaries for less disruption to the q-BIC mode and also for the quasi-radial lens to more closely approximate a radial lens, but introduce more boundary zones that deviate away from desired performance. Another option to explore is designing the lattice as slightly wedge shaped to minimize boundaries^5^.

**Section 6: Design Parameters of Experimental Devices**

| **Figure** | **Device** | **Film Thickness (nm)** | **Unperturbed Lattice Constant (nm)** | **Designed Aperture Size (nm)** | **Device Footprint (μm)** | **TE or TM mode** |
| --- | --- | --- | --- | --- | --- | --- |
| 2 | Radial lens | 125 | 450 | 125 × 375 | Diameter = 800 | TE |
| 3c | Cylindrical lens | 125 | 410 | 100 × 350 | 750 × 750 | TE |
| 3h/5b | Two-function cylindrical lens | 200 | A_x_=430, A_y_= 460 | 50 × 200 | 505 × 505 | TM |
| 4b | Quasi-radial lens | 125 | 450 | 125 × 375 | Diameter = 800 | TE |
| 4c | Two-function cylindrical lens | 100 | A_x_=430, A_y_= 460 | 50 × 250 | 505 × 505 | TE |
| 5c | Two-function cylindrical lens | 100  (covered with ~30 nm partially conformal PECVD SiO_2_) | A_x_=385, A_y_= 425 | 50 × 210 | 550 × 550 | TE |
| 6b | Single-function beam steerer at the green wavelength | TiO_2_: 25  SiO_2_: 100 | 190 | Interpolated between  30 × 170 (α=0°) and 40 × 120 (α=45°) | N/A | TE |
| 6c | Two-function beam steerer at the red and blue wavelengths | TiO_2_: 80  SiO_2_: 100 | A_x_=385, A_y_= 425 | Blue Mode: interpolated between10 × 80 (α=0°) to 10 × 77.5 (α=45°)  Red Mode: interpolated between 10 × 90 (α=0°) to 10 × 70 (α=45°) | N/A | TM |

**Table S1** Design parameters of devices in the main text.

**Section 7: Discussion on Cascaded Metasurfaces**

**Figure S12** illustrates a general workflow for cascading metasurfaces to realize multifunctionality. Any two of our nonlocal metasurfaces can be cascaded if their resonances do not overlap spectrally as they do not shape the wavefront of non-resonant light. As a parallel to our experimental cascaded nonlocal metasurfaces (**Figs. 3-5** of the main text), we consider cascading two conventional local metasurfaces without compensating for the effect of each metasurface on the other, and show that this is not a viable approach to realize multifunctionality.

**
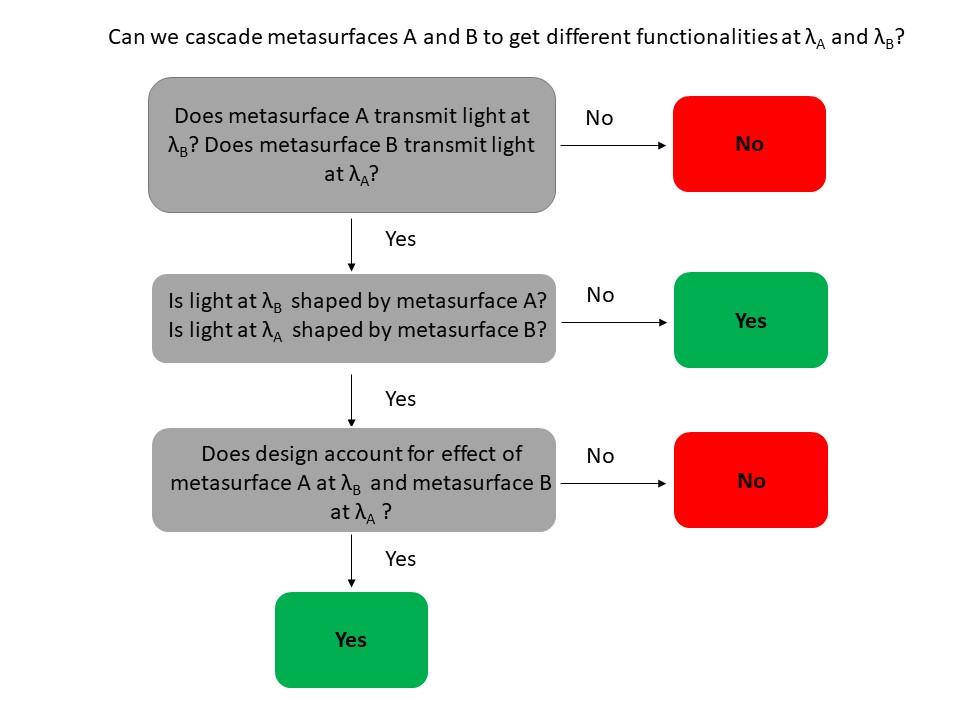
**

**Fig. S12** Flow chart outlining requirements for cascading metasurfaces.

We begin by considering metasurfaces based on conventional local geometric phase (**Fig. S13**). The meta-units for each metasurface are chosen such that both metasurfaces are highly transparent at the two wavelengths of interest, λ = 1.25 μm and 1.65 μm (dashed curves in **Fig. S13a**). In metasurfaces based on local geometric phase, a spatial phase distribution is imparted only when the circular polarization of the light is converted by the metasurface. Therefore, we devise our meta-units for one metasurface to convert minimal light at the operating wavelength of the other metasurface (solid curves in **Fig. S13a**). Note that this is only possible when there is a relatively large spectral spacing (~400 nm in the example) between the two operating wavelengths due to the broadband nature of local geometric phase.


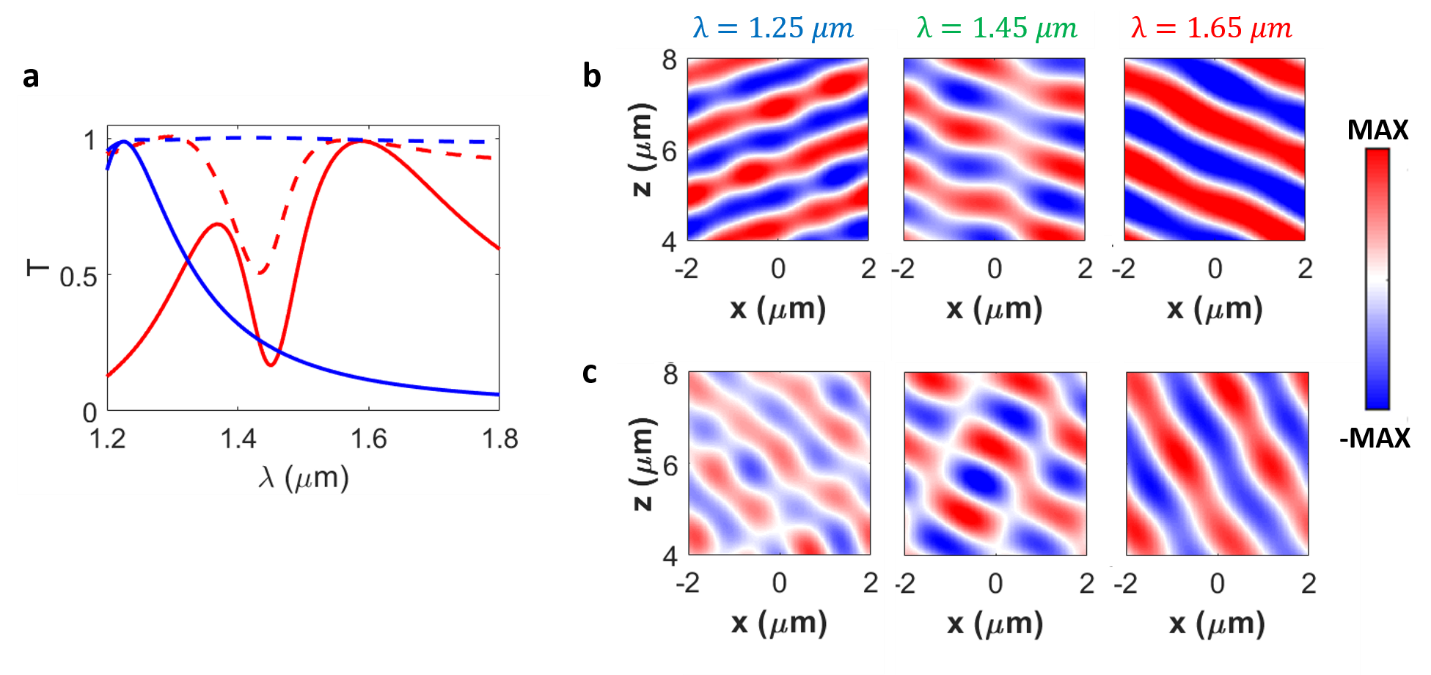


**Fig. S13** Simulations of cascaded metasurfaces based on conventional local geometric phase. (a) Total transmission spectra (dashed lines) and transmission spectra of light with converted handedness of circular polarization (solid lines) for the two phase-gradient metasurfaces, both composed of rectangular Si pillars on glass with a height of 800 nm and a lattice constant of 650 nm. ‘Red’ meta-unit pillar dimensions: W=150 nm, L=600 nm. ‘Blue’ meta-unit dimensions: W=100 nm, L=375 nm. (b-c) Simulated wavefronts (real part of electric field) for light with converted handedness (b) and unconverted handedness (c) of the doublet where the two metasurfaces are separated by a 2-μm air gap.

In this simple demonstration, we design constant phase-gradient metasurfaces at the two operating wavelengths and set up a doublet where the devices impart the same magnitude of the phase gradient but in opposite directions. The phase gradient at the ‘blue’ wavelength, λ = 1.25 μm, is $\frac{d\phi}{dx}=\frac{2\pi}{3.9 \mu m}$, and that at the ‘red’ wavelength, λ = 1.65 μm, is $\frac{d\phi}{dx}=-\frac{2\pi}{3.9 \mu m}$. The resulting simulated wavefronts are shown in **Figs. S13b,c** for handedness converted light and unconverted light, respectively. Two features are notable from the results. First, the wavefront for light of the same state of circular polarization as that of the incident light is altered by the metasurface doublet (**Fig. S13c**), whereas a single metasurface based on local geometric phase would leave the wavefront shape unchanged. This is a result of both metasurfaces imparting a phase profile at the two operating wavelengths. We note that double the magnitude of the phase gradient is applied to light of twice converted handedness (i.e., no net change of the polarization state) in this particular doublet. Second, light at intermediate wavelengths between the two operating wavelengths has its wavefront shaped (middle panels in **Figs. S13b,c**), whereas our metasurfaces based on nonlocal geometric phase leave the wavefront shape unchanged at non-resonant wavelengths.

This example illustrates the limitations of cascaded metasurfaces based on conventional local geometric phase in realizing multifunctionality: (a) The design of meta-units for one metasurface has to consider their optical response at all operating wavelengths. (b) Broadband response of meta-units prevents wavefront shaping at closely spaced operating wavelengths. (c) Optical wavefronts are deformed at wavelengths other than the chosen operating wavelengths.

Next, we consider cascading metasurfaces based on meta-units that rely on propagation phase. We simulate a meta-unit library of square silicon pillars on glass (**Fig. S14a**) and choose meta-units to form a constant phase gradient at two operating wavelengths, λ = 1.4 μm and 1.6 μm (**Fig. S14b**). We simulate these phase-gradient metasurfaces individually (**Figs. S14c,d**) and cascaded together (**Fig. S14e**). The cascaded device produces distorted wavefronts because both metasurfaces shape the wavefront at the two operating wavelengths owing to the broadband response of the meta-units. This example illustrates that it is not feasible to realize multifunctionality by simply cascading local metasurfaces based on meta-units that rely on propagation phase.


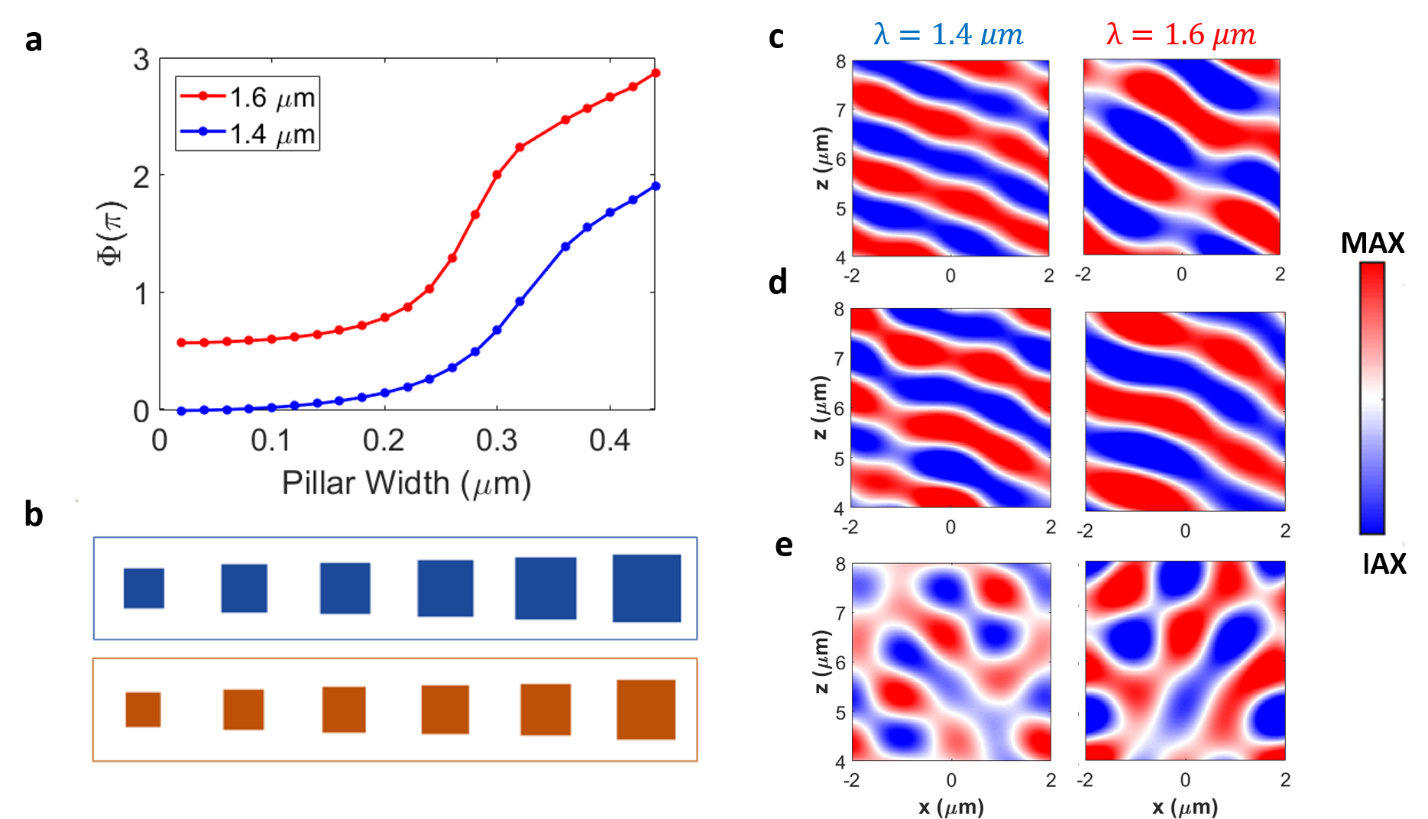


**Fig. S14** Simulation of cascaded metasurfaces based on meta-units that rely on propagation phase. (a) Simulated phase for 800 nm tall square silicon pillars on glass with a lattice constant of 650 nm at λ = 1.4 μm (blue line) and λ = 1.6 μm (red line). (b) Schematic of constant phase-gradient metasurfaces designed for a phase gradient of $\frac{d\phi}{dx}=\frac{2\pi}{3.9 \mu m}$ at a ‘blue’ wavelength of $= 1.4 m$(upper panel) and a ‘red’ wavelength of λ = 1.6 μm (lower panel). (c-e) Simulated wavefronts (real part of electric field) for the ‘blue’ metasurface (c), the ‘red’ metasurface (d), and a doublet composed of the two metasurfaces separated by a 2-μm air gap (e).

**Table S2** details cascaded metasurfaces reported in the literature, most of which differ from the above two examples in that they do account for the effect of each metasurface at each operating wavelength with the consequent design complications (i.e., design considerations in **Table S2**).

| **Work** | **Notes** | **Mechanism** | **Operating wavelengths (nm)** | **Design considerations** | **Non-selected wavelengths** |
| --- | --- | --- | --- | --- | --- |
| Ref. 6 | 3-layer, 3-color, 3-function metalenses with wavefront at each color shaped deliberately | Propagation phase: truncated Si waveguides | 1180/1400/1680 | * Each metasurface primarily controls one wavelength.  * Consider contribution of each metasurface to each wavelength.  * Lateral alignment of metasurfaces matters. | Potentially distorted wavefronts |
| Ref. 7 | Metasurface doublets with engineered chromatic dispersion | Propagation phase: truncated Si waveguides | 540/550/560 or 750-1000 | * Consider chromatic dispersion of each metasurface and propagation phase between metasurfaces.  * Alignment of metasurfaces matters. | Following designed chromatic dispersion |
| Refs. 8,9 | 2-layer, 3-color, 3-function holograms | Phase-amplitude meta-units (1^st^ element)  Propagation phase meta-units (2^nd^ element) | 1000/1300/1500 or  460/530/620 | * 1^st^ element consists of 3 separate metasurface holograms.  * 2^nd^ element combines images while compensating for chromatic dispersion.  * Alignment of metasurfaces matters. | Potentially distorted wavefronts |
| Ref. 10 | 3-layer, 3-color, 3-function metalenses with wavefront at each color shaped deliberately | Plasmonic scatterers | 450/550/650 | * Different plasmonic material for each layer targeting one resonant wavelength.  * Plasmonic resonances have to be sufficiently separated spectrally and layers must be sufficiently spatially separated to avoid crosstalk | Potentially distorted wavefronts or minimal transmission |
| Ref. 11 | 2-layer, 1-color, 1-function holograms. | Can use geometric phase or propagation phase | 740 | * Doublet encodes a distinct hologram compared to each element independently.  * Machine learning (gradient optimization) to design doublet.  * Lateral alignment of metasurfaces matters. | N/A (monochromatic doublet) |
| Ref. 12 | 3-layer, 2-color, 4-function metasurface holograms with polarization multiplexing | Plasmonic scatterers: Au nanostructures | 562/659 | * Machine learning (hybrid deep learning) to design triplet. | Potentially distorted wavefronts or minimal transmission |
| Ref. 13 | 2-layer, 1-color, 1-function cascaded metasurfaces for retroreflector | Propagation phase: truncated Si waveguides | 850 | * 1^st^ element performs Fourier transform.  * 2^nd^ element imparts constant phase gradient. | Chromatic aberration and decreased efficiency |
| **This work** | 2-layer, 4-color, 4-function metalens (**Fig. 5** of the main text) | q-BIC, geometric phase | 1388/1414/  1480/1622 | * q-BICs must not overlap spectrally. | Undistorted wavefronts |

**Table S2** Overview of current literature on cascaded metasurfaces.

**Section 8: Additional Cascaded Metalens Measurement
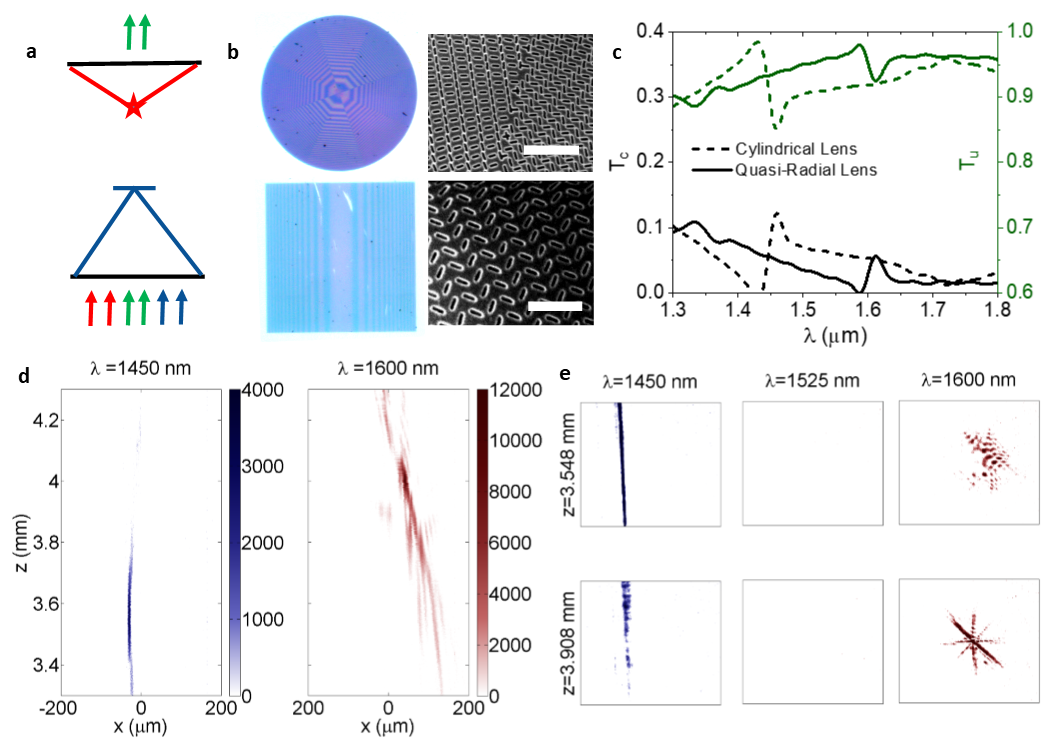
**

**Fig. S15** Experimental results of a nonlocal metalens doublet composed of a quasi-radial lens and a cylindrical lens. (a) Schematic of the operation of the doublet with incident light interacting with the cylindrical lens and the quasi-radial lens in sequence. (b) Optical and scanning electron microscopy images of the quasi-radial (top) and cylindrical (bottom) lenses. Scale bars: 2 μm. (c) Transmission spectra of light with converted circular polarization (T_c_) and unconverted circular polarization (T_u_) for the cylindrical and quasi-radial lenses. (d) Longitudinal far-field scans of transmitted light with converted circular polarization. (e) Transverse far-field scans of transmitted light with converted circular polarization.

**Section 9: Naive Spatial Multiplexing**

To emphasize that our *p1* metasurfaces (e.g., dual-function cylindrical metalenses in **Figs. 3-5** of the main text) are not simply devised by a spatial multiplexing approach but rather require symmetry degeneration, we demonstrate in simulation that simple spatial multiplexing of meta-units would not lead to multifunctionality. The spatially multiplexed supercell consists of two different *p2* meta-units on the same substrate and with the same lattice constant but different aperture geometry such that one has apertures with 70% smaller in-plane dimensions than the other. Independently, each meta-unit has a distinct resonant wavelength (**Fig. S16a**) and the expected geometric phase control at its respective resonant wavelength (**Fig. S16b**). The transmission spectra of a periodic supercell of the two *p2* meta-units however show only one q-BIC with an intermediated resonant wavelength (**Fig. S16c**). Note that conversion efficiency is lowered compared to the separate sets of meta-units. Each pair of apertures that belong to one *p2* meta-unit controls the geometric phase response directly above the pair in the propagation direction, but mostly does not control the geometric phase response directly above the other pair of apertures in the supercell (**Fig. S16d**). In short, this supercell does not enable multifunctional behavior; it simply behaves as a single *p2* meta-unit with one resonance.

**
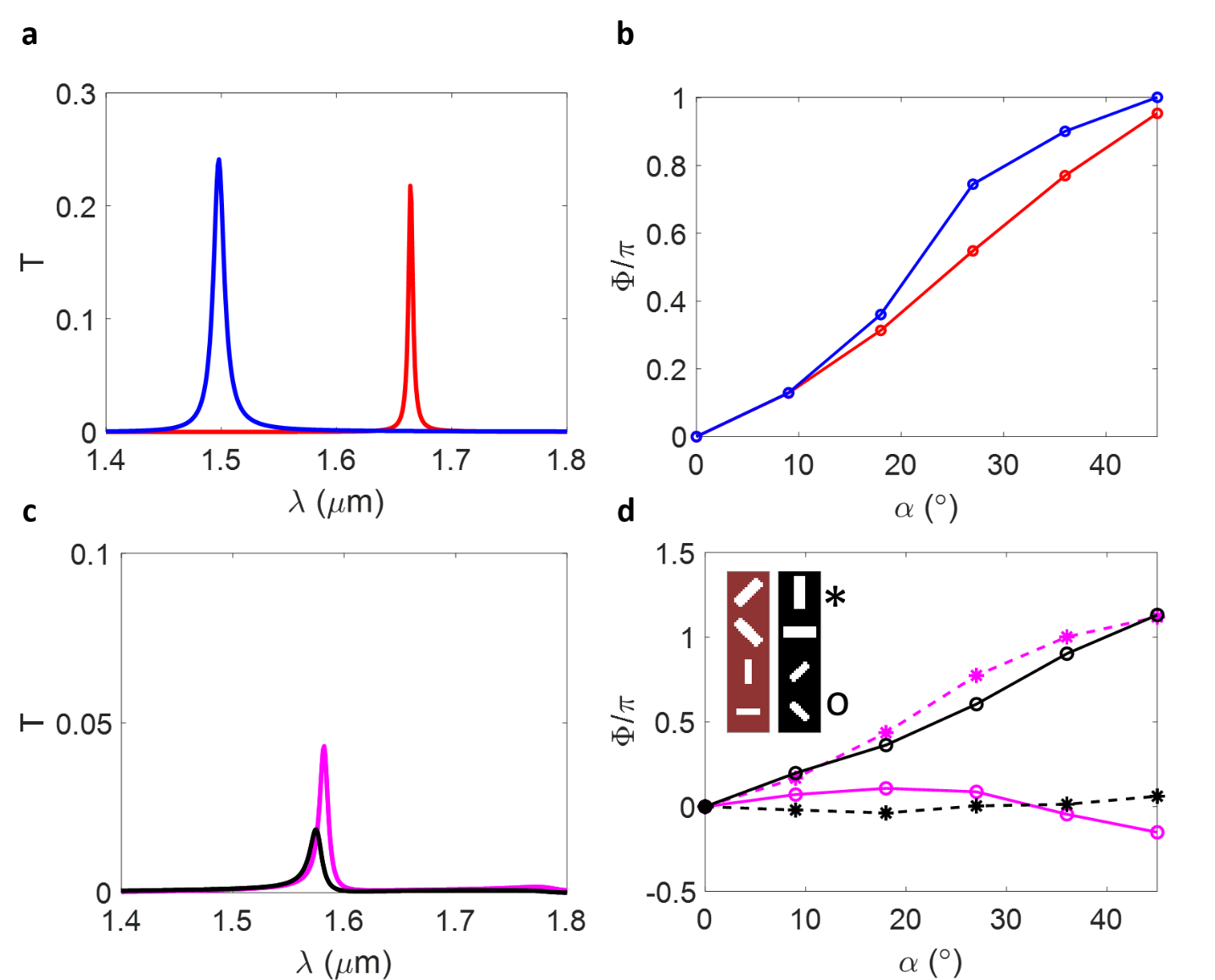
**

**Fig. S16** Simulation of a supercell of spatially multiplexed *p2* meta-units. (a) Transmission spectra of light with converted circular polarization for the *p2* meta-unit in **Fig. S3** (blue) and the same meta-unit but with the apertures scaled to 70% of their original size (red). (b) Phase response as a function of in-plane rotation angle for the meta-units in (a). (c) Transmission spectra of light with converted circular polarization for a supercell of the *p2* meta-units in (a) with periodic boundary conditions. Pink curve corresponds to the red colored inset in (d) and black curve corresponds to the black colored inset in (d). (d) Black curves: Phase responses of the supercell as a function of in-plane rotation angle of the pair of small apertures belonging to only one *p2* meta-unit above the small (circle) and large (star) apertures. Pink curves: Phase responses of the supercell as a function of in-plane rotation angle of the pair of large apertures belonging to the other *p2* meta-unit above the small (circle) and large (star) apertures. Inset: Schematics of supercells.

**Section 10: Estimate Q-factors of Cascaded Metasurfaces**

**
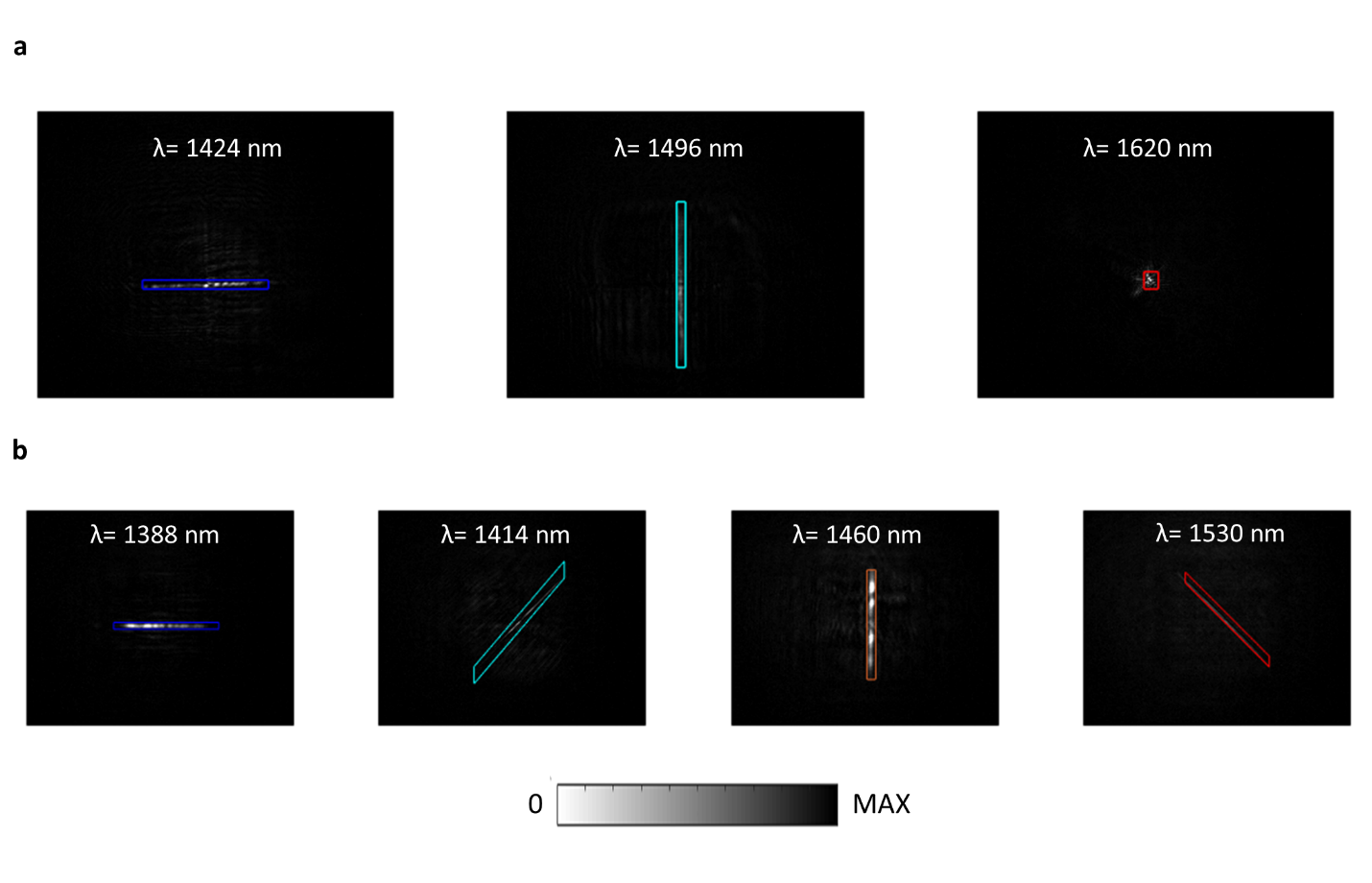
**

**Fig. S17** (a) and (b) show, respectively, cross-sectional areas used for estimating Q-factors in **Fig. 4** and **Fig. 5** of the main text. **Figures 4i** and **5g** show average intensity per pixel over a range of wavelengths spanning the resonance.

**Section 11: Multifunctional Metasurface Design and Fabrication Issues**

In principle, the resonant wavelength of TM modes with electric field components out of the plane of the device is more robust against variations in aperture sizes (**Fig. S18a**) than that of the TE modes with electric field components in the plane of the device (**Fig. S18b**). For the case of TE modes, the in-plane component of the electric field is concentrated most strongly inside the apertures (**Figs. S18c,d**). As such, variations in the size of the apertures can dramatically change the modal index and thereby severely shift the resonant wavelength. As the displacement field but not the electric field in the in-plane direction is continuous across the boundary between the etched aperture and the unetched region, filling the apertures with SiO_2_ to reduce the refractive index contrast—and therefore the electric field concentrated in the aperture—makes the resonant wavelength more robust against fabrication variations (**Fig. S18e**). In this regard, a thin film of ~30-nm SiO_2_ is deposited on the ~45° rotated device in **Fig. 5c** of the main text to help mitigate potential issues from small variations in aperture sizes across the device footprint.

**
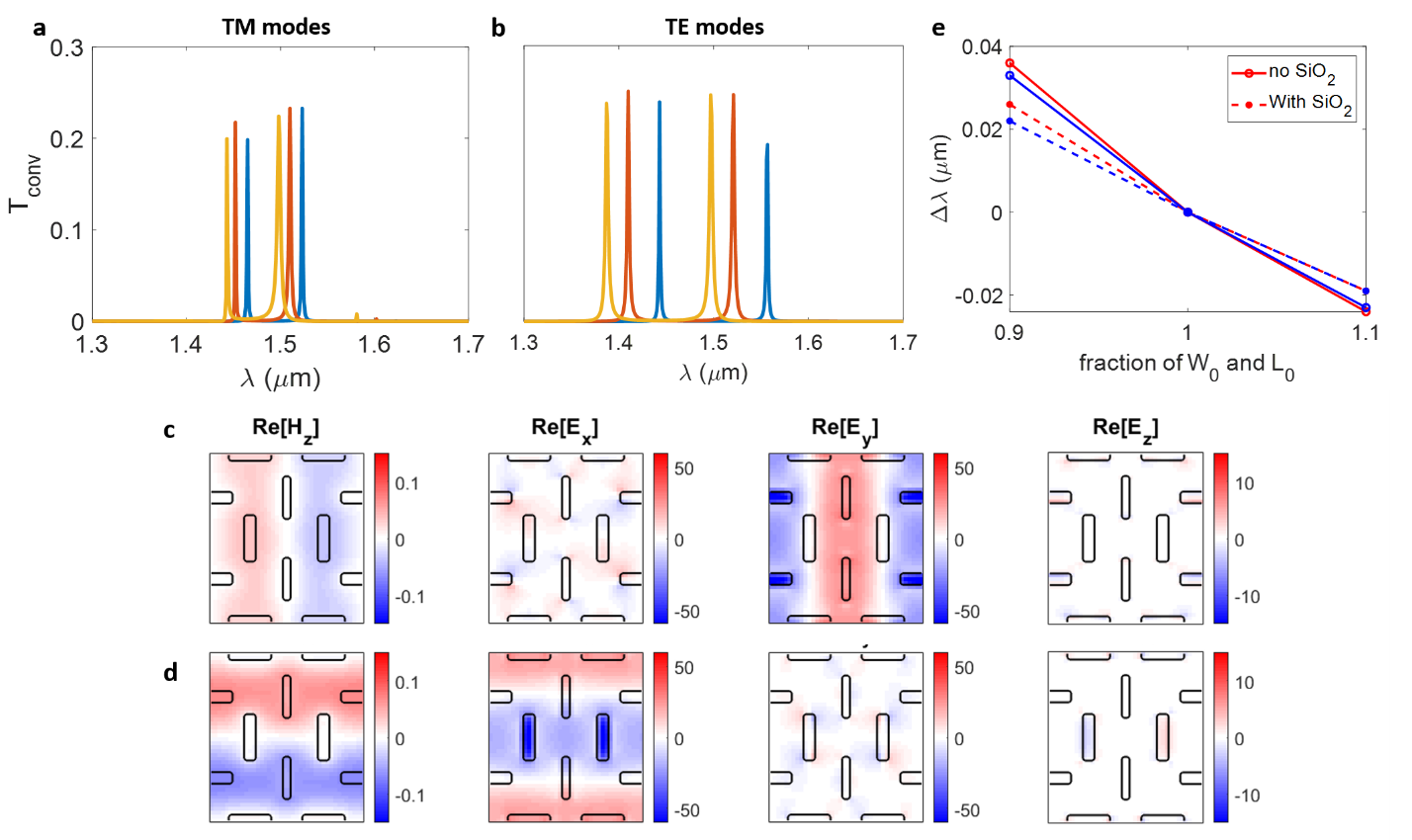
**

**Fig. S18** Simulated shift in resonant wavelengths with aperture size variation. (a) Spectra of transmitted circularly-polarized light of converted handedness of periodic meta-units with dimensions: A_x_= 430 nm, A_y_= 460 nm, and defined in a 200-nm thick a-Si thin film on glass. Red curve: aperture width W_0_= 50 nm, and aperture length L_0_= 220 nm. Blue curve: W=0.9W_0_ and L=0.9L_0_. Yellow curve: W=1.1W_0_ and L=1.1L_0_. The two resonances of each spectrum correspond to q-BIC modes with electric fields primarily out of plane (TM modes). (b) Spectra of transmitted circularly-polarized light of converted handedness of periodic meta-units with dimensions: A_x_= 425 nm, A_y_= 385 nm, and defined in a 100-nm thick a-Si thin film on glass. Red curve: aperture width W_0_=50 nm, and aperture length L_0_= 220 nm. Blue curve: W=0.9W_0_ and L=0.9L_0_. Yellow curve: W=1.1W_0_ and L=1.1L_0_. The two resonances of each spectrum correspond to q-BIC modes with primarily in-plane electric fields (TE modes). (c) Field profiles for the ‘blue’ mode at λ=1.410 μm in the red curve in (b) with apertures outlined in black. (d) Field profiles for the ‘red’ mode at λ=1.521 μm in the red curve in (b) with apertures outlined in black. (e) Variation in resonant wavelengths of the 100-nm thick meta-units in (b) (solid lines) and of the same meta-units with the apertures filled with SiO_2_ (dashed lines). Red and blue lines represent the ‘red’ and ‘blue’ modes, respectively.

Our devices must maintain a near-constant resonant wavelength across the device to function properly. For this reason, meta-units that are not robust to fabrication variations and fabrication issues that result in inconsistent meta-unit dimensions together hamper device operation. We highlight one such severe case in **Fig. S19** where substantial fabrication issues derail a multifunctional metasurface, the design of which is already not robust against aperture size variations on account of the TE modes it supports. In this device, issues with the substrate orientation in the electron beam lithography process likely resulted in a device where the aperture size varies across the device footprint (**Figs. S19a-c**). Images of circularly-polarized light with converted handedness on the device plane (**Fig. S19d**) show zone patterns suggesting that there is not a resonant wavelength at which the entire device acts as either a horizontal cylindrical lens or a vertical cylindrical lens. Instead, only a portion of the device is resonant at each excitation wavelength: the right-hand side of the horizontal cylindrical lens has a resonant wavelength around λ=1,400 nm, while the left-hand side has a resonant wavelength around λ=1,500 nm; similarly, the right-hand side of the vertical cylindrical lens has a resonant wavelength around λ=1,460 nm, but the left-hand side has a resonant wavelength around λ=1,600 nm. At λ=1,460-1,480 nm, the right-hand side of the device has zone patterns of the vertical cylindrical lens, while the left-hand side has zone patterns of a horizontal cylindrical lens. **Figure S19e** shows measured circularly-polarized light with converted handedness at the device focal plane. This device has neither an observable vertical focal line nor a horizontal focal line that extends across the device footprint at any of the excitation wavelength. The partial horizontal focal line shifts from right-hand side of the device at λ=1,400 nm to left-hand side of the device at λ=1,500 nm.

**
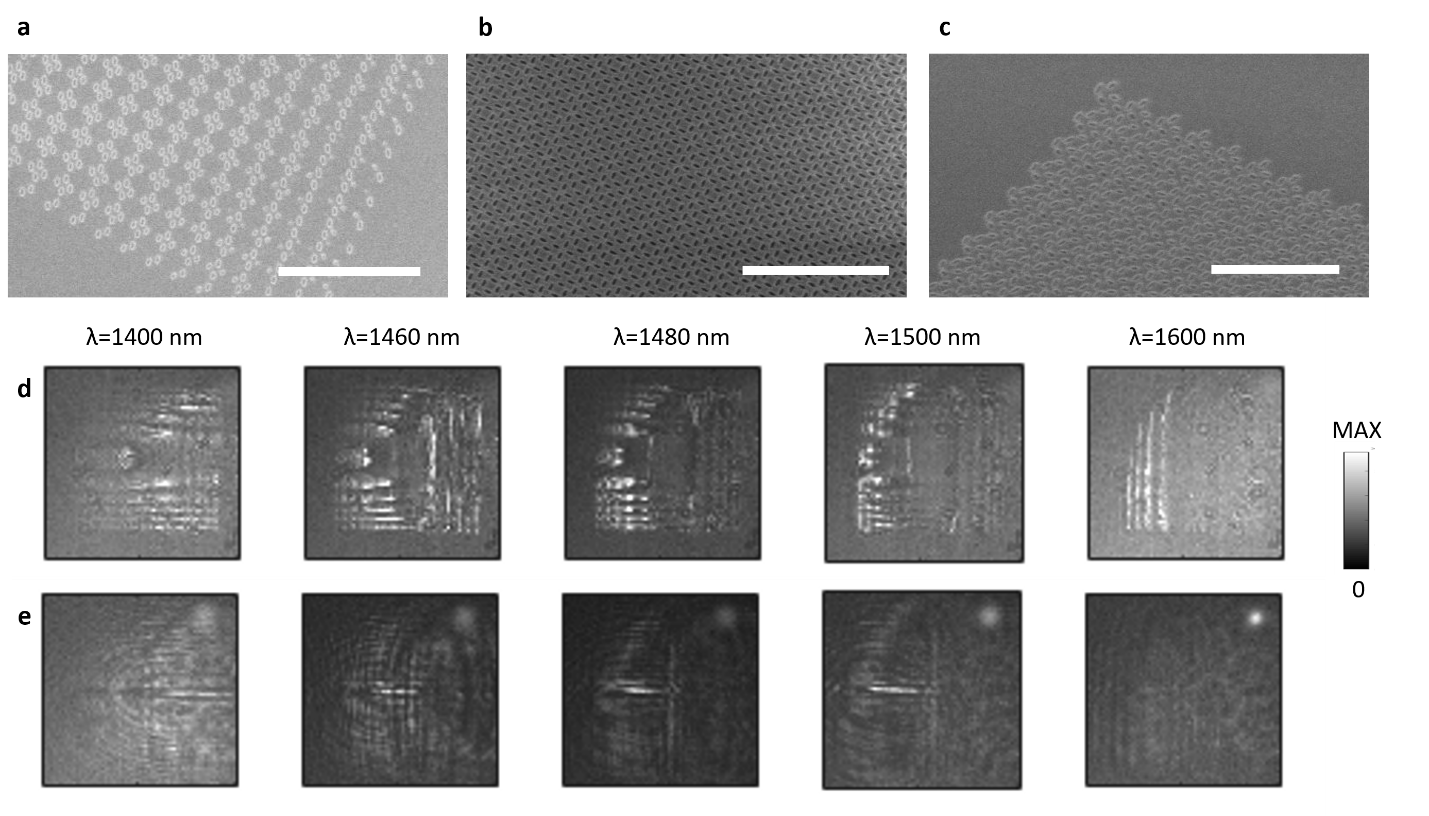
**

**Fig. S19** Measured multi-perturbation metalens performance with fabrication issues resulting in spatial aperture size variation. Device details: NA=0.06, designed dimensions A_x_=390 nm, A_y_=420 nm, W=50 nm, L=210 nm, H= 100 nm. (a-c) SEM images of the device including underexposed (a) and overexposed (c) portions of the device in diagonally opposite corners and properly exposed center of the device (b). Scale bars: 3 μm in (a) and (b), and 2 μm in (c). (d) Images of circularly-polarized light with converted handedness on the device plane at selected wavelengths. (e) Images of circularly-polarized light with converted handedness on the device focal plane at selected wavelengths.

**Section 12: Literature on Metasurface-Based Augmented Reality Solutions**

| **Work** | **Notes** | **Mechanism** | **Other Required Optical Components** | **Multiwavelength contextual information?** | **Experimental demonstration?** |
| --- | --- | --- | --- | --- | --- |
| Ref. 14 | Deflects near-IR light for eye tracking. Transparent in the visible. | Guided mode resonance | None | N/A (No contextual information) | Yes |
| Ref. 15 | Hologram with broadband high transmission | Huygen’s metasurface | Beam splitter as optical combiner | No | Yes |
| Ref. 16 | Large area RGB metalens | Geometric phase, group delay, group delay dispersion | Beam splitter as optical combiner | Yes | Yes |
| Ref. 17 | Optical see-through metalens with low efficiency and transparency | Geometric phase | Beam splitter as optical combiner, circular polarizers, dichroic mirror | Yes | Yes |
| Ref. 18 | Optical see-through metalens with low efficiency and transparency | Geometric phase | Beam splitter as optical combiner, circular polarizer at contextual information light source | No | Yes |
| Ref. 19 | Optical see-through lens doublet | Dielectric Bragg mirrors, guided mode resonance, propagation phase | None | Yes | No |
| Ref. 20 | Metalens can compensate for contour of bulk lens | Metal-Insulator-Metal | None | No | Not as see-through lens |
| **This work** | See-through metalens | q-BIC geometric phase | Circular polarizer at contextual information light source | Yes | Only near-IR |

**Table S3**: Overview of literature on free-space metasurfaces for augmented reality.

**Supplementary References**

1. Overvig, A. C., Malek, S. C. & Yu, N. Multifunctional Nonlocal Metasurfaces. *Physical Review Letters* **125**, 017402 (2020).

2. Overvig, A. C., Malek, S. C., Carter, M. J., Shrestha, S. & Yu, N. Selection rules for quasibound states in the continuum. *Physical Review B* **102**, 035434 (2020).

3. Johnson, S. G., Fan, S., Villeneuve, P. R., Joannopoulos, J. D. & Kolodziejski, L. A. Guided modes in photonic crystal slabs. *Physical Review B* **60**, 5751–5758 (1999).

4. Fan, S., Suh, W. & Joannopoulos, J. D. Temporal coupled-mode theory for the Fano resonance in optical resonators. *Journal of the Optical Society of America A* **20**, 569–572 (2003).

5. Byrnes, S. J., Lenef, A., Aieta, F. & Capasso, F. Designing large, high-efficiency, high-numerical-aperture, transmissive meta-lenses for visible light. *Optics Express* **24**, 5110–5124 (2016).

6. Zhou, Y. *et al.* Multilayer Noninteracting Dielectric Metasurfaces for Multiwavelength Metaoptics. *Nano Letters* **18**, 7529–7537 (2018).

7. McClung, A., Mansouree, M. & Arbabi, A. At-will chromatic dispersion by prescribing light trajectories with cascaded metasurfaces. *Light: Science & Applications* **9**, 1–9 (2020).

8. Huang, X., Shrestha, S., Overvig, A. & Yu, N. Three-Color Phase-Amplitude Holography with a Metasurface Doublet. Proceedings of 2020 Conference on Lasers and Electro-Optics (CLEO). San Jose, CA, USA: IEEE, 2020.

9. Overvig, A., Shrestha, S., Malek, S. & Yu, N. Artifact-free Phase-Amplitude Metasurface Holography at up to Three Wavelengths. Proceedings of 2019 Conference on Lasers and Electro-Optics (CLEO). San Jose, CA, USA: IEEE, 2019.

10. Avayu, O., Almeida, E., Prior, Y. & Ellenbogen, T. Composite functional metasurfaces for multispectral achromatic optics. *Nature Communications* **8**, 1–7 (2017).

11. Georgi, P. *et al.* Optical secret sharing with cascaded metasurface holography. *Science Advances* **7**, eabf9718 (2021).

12. Zhu, D., Liu, Z., Raju, L., Kim, A. S. & Cai, W. Building Multifunctional Metasystems via Algorithmic Construction. *ACS Nano* **15**, 2318–2326 (2021).

13. Arbabi, A., Arbabi, E., Horie, Y., Kamali, S. M. & Faraon, A. Planar metasurface retroreflector. *Nature Photonics* **11**, 415–420 (2017).

14. Song, J.-H., van de Groep, J., Kim, S. J. & Brongersma, M. L. Non-local metasurfaces for spectrally decoupled wavefront manipulation and eye tracking. *Nature Nanotechnology* **16**, 1224–1230 (2021).

15. Song, W. *et al.* Large-Scale Huygens’ Metasurfaces for Holographic 3D Near-Eye Displays. *Laser & Photonics Reviews* **15**, 2000538 (2021).

16. Li, Z. *et al.* Meta-optics achieves RGB-achromatic focusing for virtual reality. *Science Advances* **7**, eabe4458 (2021).

17. Lee, G.-Y. *et al.* Metasurface eyepiece for augmented reality. *Nature Communications* **9**, 1–10 (2018).

18. Lan, S. *et al.* Metasurfaces for Near-Eye Augmented Reality. *ACS Photonics* **6**, 864–870 (2019).

19. Bayati, E. *et al.* Design of achromatic augmented reality visors based on composite metasurfaces. *Applied Optics* **60**, 844–850 (2021).

20. Nikolov, D. K. *et al.* Metaform optics: Bridging nanophotonics and freeform optics. *Science Advances* **7**, eabe5112 (2021).
